# Direct Lithium Extraction from α-Spodumene through Solid-State Reactions for Sustainable Li_2_CO_3_ Production

**DOI:** 10.1021/acs.inorgchem.4c01722

**Published:** 2024-07-09

**Authors:** Shilong Wang, Nathan J. Szymanski, Yuxing Fei, Wenming Dong, John N. Christensen, Yan Zeng, Michael Whittaker, Gerbrand Ceder

**Affiliations:** † Department of Mat. Sci. & Engineering, UC Berkeley, Berkeley, California 94720, United States; ‡ Materials Sciences Division, Lawrence Berkeley National Laboratory, Berkeley, California 94720, United States; § Energy Geosciences Division, Lawrence Berkeley National Laboratory, Berkeley, California 94720, United States; ∥ Department of Chemistry & Biochemistry, Florida State University, Tallahassee, Florida 32306, United States; ⊥ Department of Earth & Planetary Sci., UC Berkeley, Berkeley, California 94720, United States

## Abstract

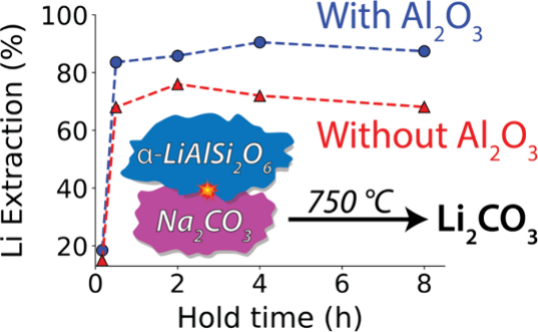

With increasing battery demand comes a need for diversified Li sources beyond brines. Among all Li-bearing minerals, spodumene is most often used for its high Li content and natural abundance. However, the traditional approach to process spodumene is costly and energy-intensive, requiring the mineral be transformed from its natural α to β phase at >1000 °C. Acid leaching is then applied, followed by neutralization to precipitate Li_2_CO_3_. In this work, we report an alternative method to extract Li directly from α-spodumene, which is performed at lower temperatures and avoids the use of acids. It is shown that Li_2_CO_3_ is formed with >90% yield at 750 °C by reacting α-spodumene with Na_2_CO_3_ and Al_2_O_3_. The addition of Al_2_O_3_ is critical to reduce the amount of Li_2_SiO_3_ that forms when only Na_2_CO_3_ is used, instead providing increased thermodynamic driving force to form NaAlSiO_4_ and Li_2_CO_3_ as the sole products. We find that this reaction is most effective at 4 h, after which volatility limits the yield. Following its extraction, Li_2_CO_3_ can be isolated by washing the sample using deionized water. This energy-saving and acid-free route to obtain Li_2_CO_3_ directly from spodumene can help meet the growing demand for Li.

## Abstract 2

Li_2_CO_3_ is a critical precursor for cathode synthesis which we show can be extracted directly from α-spodumene (LiAlSi_2_O_6_) through solid-state reactions with Na_2_CO_3_ and Al_2_O_3_.

## Introduction

The demand for Li is experiencing a rapid surge, showing a 27% increase in 2023 alone.^1^ As much as 80% of this demand originates from the battery market,^2^ which has been stimulated by the widespread adoption of electric vehicles and portable electronics. As a result, predictions forecast that annual Li demand (as Li_2_CO_3_ equivalent) may exceed 2 Mtons by the year 2030.^3^ To meet the growing demand, an ample and reliable supply of Li is needed. Currently, most Li-ion cathodes are synthesized from Li_2_CO_3_ or LiOH, which can each be obtained by processing brines or solid minerals. Of these two sources, solid Li minerals are more uniformly distributed across the globe while Li-rich brine deposits are heavily concentrated in Argentina, Chile, and Bolivia.^4−6^ More and more deposits of spodumene (LiAlSi_2_O_6_), the most prevalent and Li-rich mineral, have become available around the world. Its abundance, coupled with the fact that spodumene has a higher Li content than brines, creates the potential for a rich source of Li at low cost.

Spodumene is typically found in a dense monoclinic structure of the clinopyroxene type, often referred to as α-spodumene.^7^ To extract Li from this structure and produce a compound (often Li_2_CO_3_ or LiOH) that is useful for Li-ion cathode synthesis, most traditional methods first transform α-spodumene into its high-temperature tetragonal polymorph (denoted as β-spodumene) at temperatures exceeding 1000 °C.^7^ This energy-intensive heating step is generally considered to be necessary since β-spodumene has lower density and is therefore more amenable to chemical attack. Acid digestion can then be performed using concentrated sulfuric acid at temperatures of around 250 °C, resulting in the formation of Li_2_SO_4_. Soda ash (Na_2_CO_3_) is typically added to the solution after acid leaching to precipitate Li_2_CO_3_, which can be separated from the remaining byproducts by washing the entire sample with water.^8,9^

Recently, some progress has been made in improving upon the traditional approach to Li extraction from spodumene. Several published reports (listed in Table 1) have demonstrated that Li can be extracted from β-spodumene through solid-state reactions as opposed to acid leaching. For example, Gustavo et al. reacted β-spodumene with NaF for 1 h at 600 °C to obtain LiF, which enables Li recovery at a rate of 88.2%.^10^ Grasso et al. tested two different reactions with β-spodumene, including NaOH at 300 °C^11^ and Na_2_CO_3_ at 400 °C.^12^ They successfully extracted Li in the form of Li_2_CO_3_, with recovery rates of 91% and 86% after 5 and 10 h of heating with NaOH and Na_2_CO_3_, respectively. There have also been reports of Li extraction directly from α-spodumene, as opposed to the β-polymorph, thereby eliminating the need for high temperatures (>1000 °C) in the initial phase transformation step. For instance, Shihua et al. tested several Na-based reactants and found that NaOH was most effective in extracting Li from α-spodumene, leading to a recovery rate of 71% after leaching the sample with water.^13^ This was further increased by 17% to a total yield of 88% by extracting remaining Li from the residual with an acid solution. However, each set of observed products did not contain Li_2_CO_3_ or LiOH, which are generally needed for battery synthesis. They instead contained a mixture of Na_2_SiO_3_, Na_4_SiO_4_, NaAlO_2_, and Li_3_NaSiO_4_. Huidong et al. similarly reacted Na_2_CO_3_ with α-spodumene at 1100 °C for 30 min and obtained Li_2_SiO_3_ with a Li recovery rate of 95.9%.^14^ Solution-based approaches^15,16^ have also been tested for the extraction of Li from α-spodumene, yet these processes consistently form silicates such as Li_2_SiO_3_. This compound cannot be readily used for battery production without first isolating Li from the silicate anion, which typically requires acid leaching. It has been reported that Li silicate formation resulting from the reaction between Li_2_CO_3_ and SiO_2_ is nearly completed within a short time frame (≤5 h) at 800 °C, suggesting that lower temperatures and short hold times are needed to retain Li in the form of Li_2_CO_3_ after extracting it from spodumene.^17^

**Table 1:** A Summary of Recent Work That Has Made Progress in Extracting Li from Spodumene through Solid-State Reactions Involving Na Precursors^a^

spodumene polymorph	Na precursor	temp. (°C)	Li product	separation technique	efficiency
β	NaF^10^	600	LiF	leaching with H_2_O and H_2_SO_4_	88.2%
	NaOH^11^	300	Li_2_CO_3_	leaching with H_2_O (60 °C)	91%
	Na_2_CO_3_^12^	400	Li_2_CO_3_	leaching with H_2_O (60 °C)	86%
α	NaOH^13^	320	Li salt	leaching with H_2_O (80 °C) and 6 M H_2_SO_4_	88% (acid) 71% (water)
	Na_2_CO_3_^13^	851	Li salt	leaching with H_2_O (80 °C) and 6 M H_2_SO_4_	76% (acid) 27% (water)
	Na_2_CO_3_^14^	1100	Li_2_SiO_3_	leaching with NaOH and precipitating with Na_2_CO_3_	95.9%
	Na_2_CO_3_^18^	750	Li_2_CO_3_	removing silicates with CaO and precipitating Li_2_CO_3_ with flowing CO_2_	91.4%

a The temperature values provided refer to the conditions at which the solid-state reactions were carried out. The Li products of each reaction are listed, and in cases where the identify of these products are not reported, “Li salt” is written. After isolation of the Li product using the specified separation technique, the reported efficiency refers to the percentage of Li that was successfully extracted from spodumene

In this work, we show that Li_2_CO_3_ can be formed with a yield >90% by directly reacting α-spodumene with Na_2_CO_3_ and Al_2_O_3_. This solid-state reaction can be performed at a relatively low temperature (compared to the α- to β-spodumene transformation) of 750 °C and with a short hold time of 4 h. To clarify the role of each reactant in extracting Li from α-spodumene, we first apply *in situ* XRD to characterize its reaction with Na_2_CO_3_ (excluding Al_2_O_3_). The resulting data shows the formation of nepheline (NaAlSiO_4_) and Li_2_SiO_3_ above 600 °C, consistent with previous reports.^12^ Washing the product with water reveals a third product, Li_2_CO_3_, but in quantities that suggest only 76% of Li is extracted from spodumene when Na_2_CO_3_ is used as the sole reactant. The limited yield of Li in the form of Li_2_CO_3_ is attributed to the competing formation of Li_2_SiO_3_. To avoid this byproduct and improve the purity of Li_2_CO_3_, we identify Al_2_O_3_ as an effective additive which traps the silicate anions from spodumene and provides increased thermodynamic driving force to form Li_2_CO_3_. The reaction with Na_2_CO_3_ and Al_2_O_3_ was tested experimentally, and its products were characterized using XRD and Inductively Coupled Plasma Mass Spectrometry (ICP-MS). These measurements reveal that 90.4% of Li is successfully extracted from spodumene in the form of Li_2_CO_3_, which can be isolated using a low-cost washing procedure without acid. We propose that this approach can become an environmentally friendly method of using domestically sourced α-spodumene to create Li-salts for the battery industry.

## Results

### Characterization of Spodumene Concentrate

**Figure 1:**
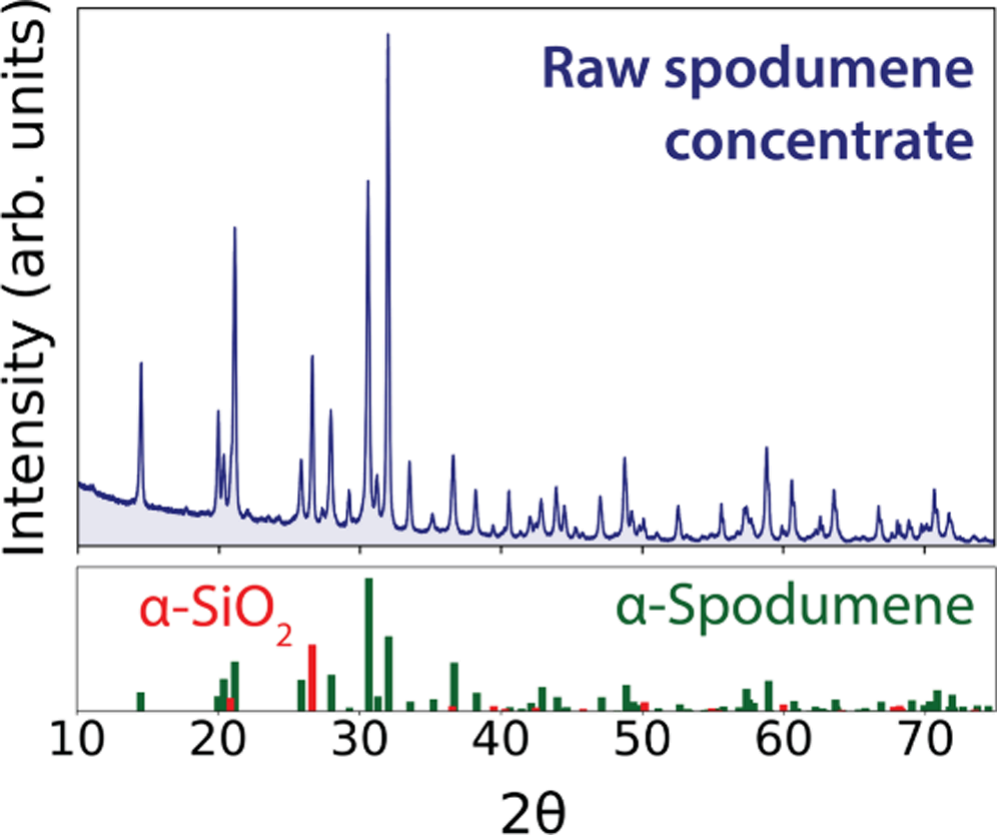
XRD pattern (Cu Kα) of raw spodumene concentrate obtained from Piedmont Lithium. Reference patterns for the two most predominant phases α-spodumene (ICSD #9668) and α-SiO_2_ (ICSD #100341) are shown in the lower panel.

The spodumene ore used in this study was provided by Piedmont Lithium Inc., who sourced it from the Carolina Tin-Spodumene Belt (TSB) in North Carolina. Previous studies have reported that the ore mined from this area generally contains 20% spodumene, 30% quartz, 43% feldspar, and 5% mica.^19^ There also tends to be trace amounts of biotite, calcite, pyrite, chlorite, apatite, and other silicates present.^20^ Piedmont Lithium Inc. purified the ore by using a combination of physical separation, size reduction, floatation, and magnetic separation. We then ground and filtered the resulting concentrate to obtain particles <75 μm in diameter by passing the sample through a 200 mesh. Scanning Electron Microscopy (SEM) confirms that the sieved concentrate contains particles with size ranging from 5 to 75 μm (Figure S1). The XRD pattern obtained from this sample is shown in Figure 1, and Rietveld refinement (Figure S2) indicates that α-spodumene is the majority phase, comprising 69.6 ± 0.14% of the sample’s weight. Three impurities are also identified: quartz (SiO_2_, 18.8 ± 0.06%), feldspar (NaAlSi_3_O_8_, 9.4 ± 0.09%), and lepidolite mica (K(Li,Al)_3_(Al,Si,Rb)_4_O_10_(F,OH)_2_, 2.1 ± 0.01%). These results are consistent with previous work, in which quartz and feldspar are reported to accompany spodumene.^7^ Related studies have also shown the prevalence of lepidolite distributed in pegmatite minerals, from which spodumene is sourced.^21,22^ A spodumene weight fraction of 69.6% corresponds to a Li_2_O content of 5.59% in our sample, which aligns well with previous reports on the Li_2_O content in spodumene, generally ranging from 5.0% to 7.2%.^13,15,20^ Our ICP-MS measurements produce a similar value of 6.18% Li_2_O in the spodumene concentrate (Table 2), and this value is used to determine the Li yield reported in later sections.

**Table 2:** Weight Fraction of Each Elemental Oxide Group That Exists in Spodumene Concentrate, as Determined by ICP-MS Measurements

	Li_2_O	Al_2_O_3_	SiO_2_	Na_2_O	K_2_O	Fe_2_O_3_	CaO
weight %	6.18	23.10	66.80	1.34	0.38	0.68	1.00

### Reaction of α-Spodumene with Na_2_CO_3_

We first studied the reaction that occurs between α-spodumene and Na_2_CO_3_ without introducing any other additives. These reactants were mixed in a 2:1 molar ratio to match the stoichiometry needed to form Li_2_CO_3_ and nepheline, according to the chemical equation below:
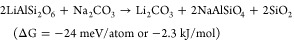
R1

The reaction energy (Δ*G*) was determined using ab initio calculations and normalized by the total number of atoms (or moles of atoms) in the products formed, as detailed in the Methods. The reactant mixture was packed in a sapphire capillary and heated to 800 °C in air at a rate of 10 °C/min. The heating process was monitored separately using *in situ* XRD measurements at the Advanced Light Source (ALS) powder diffraction Beamline 12.2.2, where the sample was scanned four times each minute (Methods).

**Figure 2:**
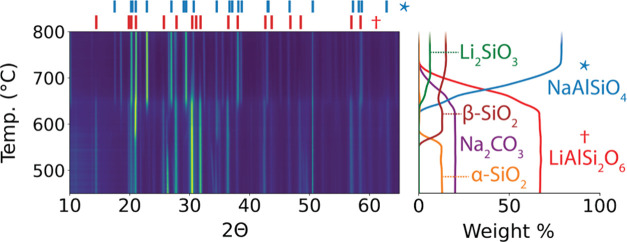
*In situ* XRD studies of the solid-state reaction between α-spodumene and Na_2_CO_3_. The heatmap in the left panel represents intensities obtained from synchrotron XRD measurements applied *in situ* while heating. The values of 2θ shown on the *x*-axis of the heatmap were obtained by converting the synchrotron wavelength (λ = 0.4959 *A*°) into Cu Kα (λ = 1.5406 *A*°) for ease of analysis. In the right panel, we plot the weight fraction of each phase as a function of temperature.

The heatmap shown in the left panel of Figure 2 displays XRD intensities that were collected from the sample containing α-spodumene and Na_2_CO_3_ as it was heated to 800 °C. Reaction products were identified with the aid of XRD-AutoAnalyzer,^23^ and their weight fractions were determined using Rietveld refinement. Only crystalline phases were accounted for, neglecting the possible formation of amorphous byproducts that are often prevalent in Si-containing systems but difficult to identify using XRD alone. The weight fraction of each crystalline phase is plotted as a function of temperature in the right panel of Figure 2. These results show that α-spodumene and Na_2_CO_3_ begin to react at 600 °C, forming nepheline (NaAlSiO_4_) as the majority product. It is anticipated that Li_2_CO_3_ forms in addition to nepheline; however, Li_2_CO_3_ is difficult to detect in multiphase XRD patterns owing to its weak scattering of X-rays, and therefore we do not attempt to quantify its weight fraction without first isolating it from the other compounds. In contrast, a minority Li_2_SiO_3_ phase is clearly detected shortly after the formation of nepheline. The weight fractions of both phases continue to grow until 750 °C, at which point spodumene is mostly consumed. No further changes are observed upon heating the sample above 750 °C. Throughout all the temperatures we sampled, there is little change to the amount of SiO_2_ that exists in the starting material. Though, it does exhibit a well-established phase transition from its α to β polymorph in the range of 550–600 °C.^24^

The observed formation of Li_2_SiO_3_ suggests that not all Li was successfully extracted from spodumene in the form of Li_2_CO_3_, which itself is difficult to detect from XRD alone. The origin of Li_2_SiO_3_ can be found by inspecting the proposed reaction (R1), which shows that any Li_2_CO_3_ formed by reacting Na_2_CO_3_ with spodumene must also be accompanied by the formation of SiO_2_ to balance the chemical equation—i.e., the silicate anions from spodumene cannot be accommodated by nepheline alone. DFT computations indicate that a reaction between Li_2_CO_3_ and SiO_2_ becomes thermodynamically favorable at temperatures above 400 °C, and this finding is consistent with the fact that Li_2_SiO_3_ does indeed form shortly after the appearance of nepheline at 600 °C (Figure 2). These results are also in agreement with previous findings, as Grasso et al. reported the simultaneous formation of NaAlSiO_4_ and Li_2_SiO_3_ at 560 °C when β-spodumene reacts with Na_2_CO_3_.^12^ Despite the presence of Li_2_SiO_3_ in our sample, its low weight fraction (∼6.0%) relative to that of NaAlSiO_4_ (∼79.1%) suggests that Li_2_SiO_3_ contains only about 24.1% of the Li that was extracted from spodumene. We will show in the next section that much of the remaining (unaccounted for) Li is present in the form of Li_2_CO_3_.

### Separation of Li_2_CO_3_ from NaAlSiO_4_

The reaction of α-spodumene with Na_2_CO_3_ produces a mixture of NaAlSiO_4_ and Li_2_SiO_3_, with Li_2_CO_3_ anticipated as a potential byproduct. Detecting Li_2_CO_3_ directly is made difficult by the fact that it scatters X-rays weakly, exhibits strong peak overlap with NaAlSiO_4_, and likely has a low weight fraction in the sample compared to the heavier NaAlSiO_4_ phase. To validate that Li_2_CO_3_ is present, we separated it from the other phases by leveraging their solubility differences in water. Li_2_CO_3_ is reported to have a solubility of 1.30 g per 100 g of water (pH = 7) at 25 °C,^25^ whereas NaAlSiO_4_, Li_2_SiO_3_, and SiO_2_ are poorly soluble under the same conditions. The separation process used here follows a similar procedure to the methods outlined in previous studies, where Li_2_CO_3_ was isolated from several aluminosilicate phases using water.^11,12^ In the current work, we added the products to DI water, with the assistance of sonication to reduce particle agglomeration. The solution was then stirred thoroughly for 1.5 h to ensure complete dissolution of Li_2_CO_3_, followed by filtering of the sample through an inorganic membrane with a pore size of 0.1 μm. The filtered liquid was placed in an oven and kept at 70 °C overnight to evaporate water and isolate the solid. This solid was characterized using XRD measurements, and its Li concentration was determined using ICP-MS measurements.

**Figure 3:**
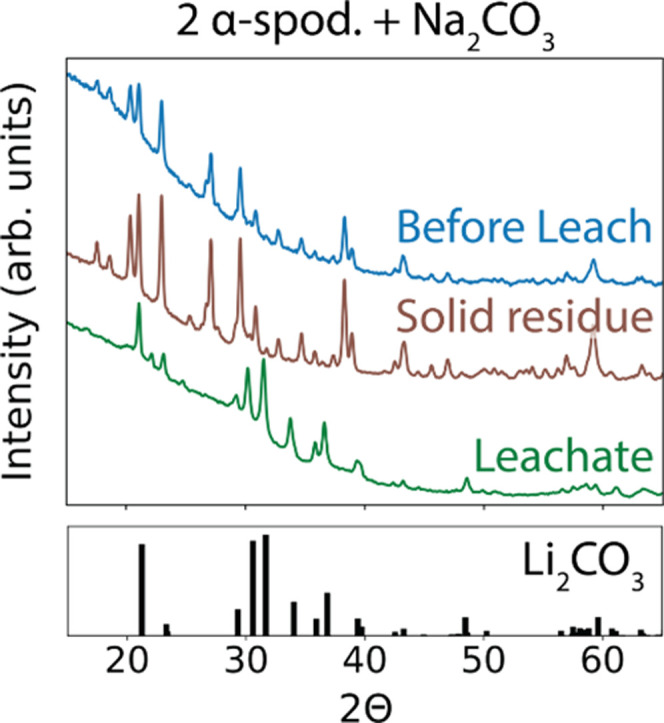
XRD patterns (Cu Kα) of the products that were obtained by reacting α-spodumene with Na_2_CO_3_. The data is shown for the sample before and after leaching with water. The washing procedure results in a solid residue (not dissolved) and a leachate which is dried and characterized. The pattern of this leachate confirms the presence of Li_2_CO_3_ (ICSD #66941), whose reference peaks are shown in the lower panel.

The XRD patterns collected from the sample before and after the washing procedure are shown in Figure 3. Before washing, the XRD pattern contains peaks from both NaAlSiO_4_ and Li_2_SiO_3_, while the suspected peaks from Li_2_CO_3_ are difficult to detect. After washing, the XRD pattern from the solid residue (that was not dissolved in water) appears similar to the original pattern, suggesting that NaAlSiO_4_ and Li_2_SiO_3_ remain inert in this treatment. The XRD pattern obtained from the leachate (that was dissolved in water and then dried) matches well with the reference pattern for Li_2_CO_3_, as shown in Figure 3. Rietveld refinement also confirms Li_2_CO_3_ as a majority phase (90 ± 0.2%) in the sample (Figure S3). The rest of the sample appears to be comprised of SiO_2_ (10 ± 0.3%). This indicates that the washing procedure is effective in isolating Li_2_CO_3_ from the other solid byproducts that formed from the solid-state reaction between α-spodumene and Na_2_CO_3_. Despite its success, ICP-MS measurements performed on the leachate reveal that only 69% of Li was successfully extracted from spodumene and recovered in the form of Li_2_CO_3_. This finding is consistent with our earlier observation that some of the Li from spodumene instead formed Li_2_SiO_3_, which has a low solubility in water and is less useful for battery manufacturing.

### Introducing Al_2_O_3_ as an Additive

We have shown in the previous sections that Na_2_CO_3_ can extract Li from α-spodumene through a solid-state reaction which forms Li_2_CO_3_, Li_2_SiO_3_, NaAlSiO_4_, and SiO_2_. Isolation of Li_2_CO_3_ can then be achieved through a washing process, but only with a moderate (69%) yield of Li as determined by ICP-MS. Avoiding the formation of Li_2_SiO_3_ is key to increasing this yield further. For this task, we introduce Al_2_O_3_ as an additive that participates in the solid-state reaction between α-spodumene and Na_2_CO_3_. The choice of Al_2_O_3_ is motivated by two anticipated benefits. First, its addition compensates for the excess silicate anions in spodumene and reduces the likelihood of Li_2_SiO_3_ formation. This means that Li_2_CO_3_ can now form as the sole byproduct of NaAlSiO_4_ based on the following reaction stoichiometry:
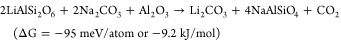
R2

Second, this reaction has a much larger thermodynamic driving force than the reaction between Na_2_CO_3_ and spodumene (without Al_2_O_3_). The change in the free energy (Δ*G*) associated with forming Li_2_CO_3_ increases from −24 meV/atom (R1) to −95 meV/atom (R2) after Al_2_O_3_ is introduced as an additive.

**Figure 4:**
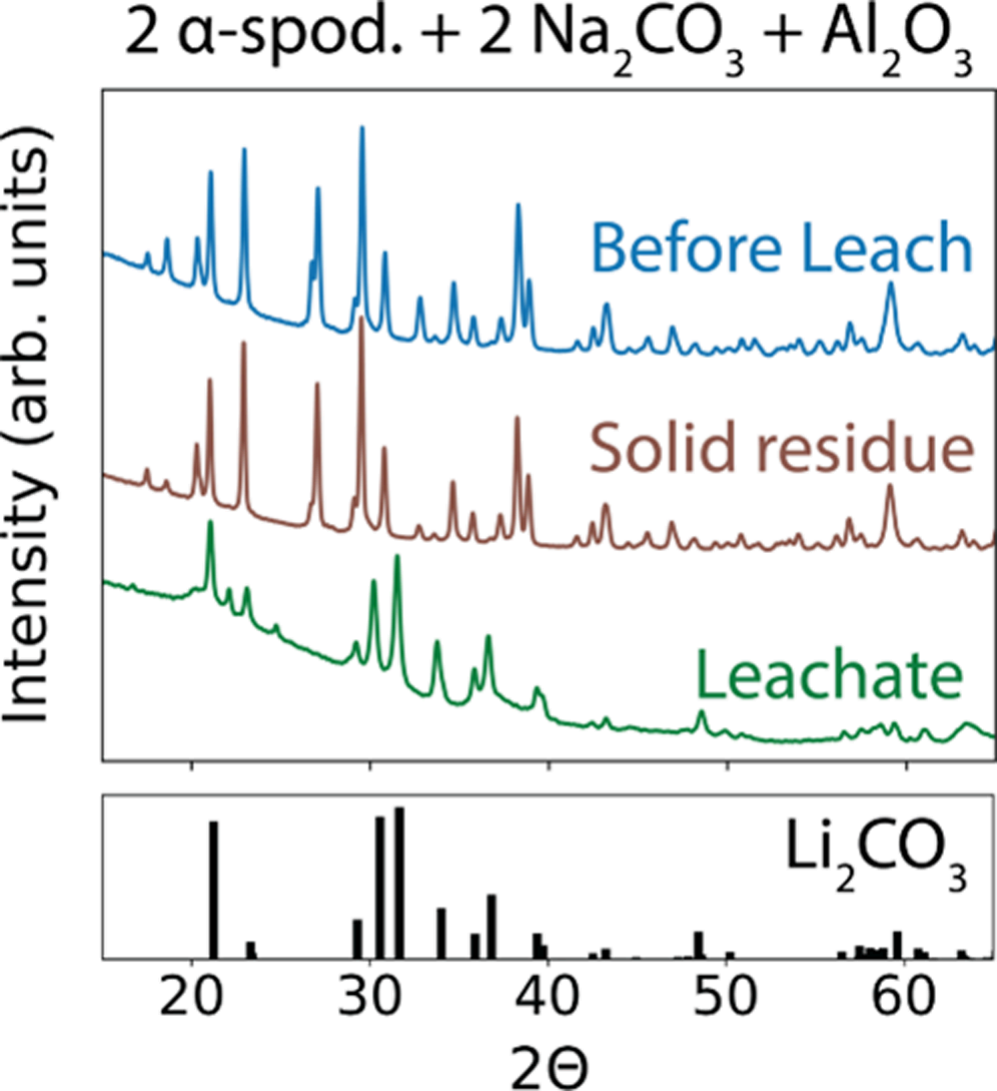
XRD patterns (Cu Kα) of the products that were obtained by reacting α-spodumene with Na_2_CO_3_ and Al_2_O_3_. The data is shown for the sample before and after leaching with water. The washing procedure results in a solid residue (not dissolved) and a leachate which is dried and characterized. The pattern of this leachate confirms the presence of Li_2_CO_3_ (ICSD #66941), whose reference peaks are shown in the lower panel.

To validate the effectiveness of the newly proposed reaction (R2), we mixed α-spodumene with Na_2_CO_3_ and Al_2_O_3_ in a 2:2:1 molar ratio and heated the sample to 750 °C for 30 min. After letting the sample cool to room temperature, it was manually ground and washed with water using the same procedure outlined in the previous section. The XRD patterns obtained from the sample before and after washing with water are shown in Figure 4. These results show that the sample obtained directly after synthesis contains a predominant NaAlSiO_4_ phase. XRD performed on the solid residue (after removing Li_2_CO_3_) also reveals a Li_2_SiO_3_ byproduct, but Rietveld refinement suggests that its weight fraction is nearly cut in half (decreasing from 6.0% to 3.1%) relative to the sample prepared using Na_2_CO_3_ alone (Figures S4–S6). What little Li_2_SiO_3_ does form likely originates from reactions with the SiO_2_ phase that coexists with spodumene. As shown in Figure 1, the spodumene concentrate used in this study contains a prominent SiO_2_ impurity with a weight fraction of 18.8%. Therefore, although Al_2_O_3_ circumvents the formation of additional SiO_2_ during the extraction of Li from spodumene, it does not prevent any reactions that may occur between Li_2_CO_3_ (extracted from spodumene) and SiO_2_ (that is already present in the sample).

Despite the presence of Li_2_SiO_3_ in the sample, its reduced weight fraction points to the success of the proposed reaction (R2) and suggests that more Li from spodumene successfully contributed to the formation of Li_2_CO_3_ instead of becoming trapped in Li_2_SiO_3_. Enhanced Li_2_CO_3_ formation is further evidenced by the results from ICP-MS measurements performed on the leachate, which reveals that 85% of all Li from spodumene is present in the form of Li_2_CO_3_. This represents a 16% increase in yield compared to the sample prepared without Al_2_O_3_. Although both samples (made with and without Al_2_O_3_) display a strong diffuse background in XRD, which may be caused by the presence of amorphous impurities that likely contain Si, confirmation of the high Li yield with ICP-MS suggests that such impurities have little effect on Li_2_CO_3_ formation. Further improvements can also be achieved with longer hold time at 750 °C, as demonstrated in the next section.

### Optimizing the Yield of Li_2_CO_3_

**Figure 5:**
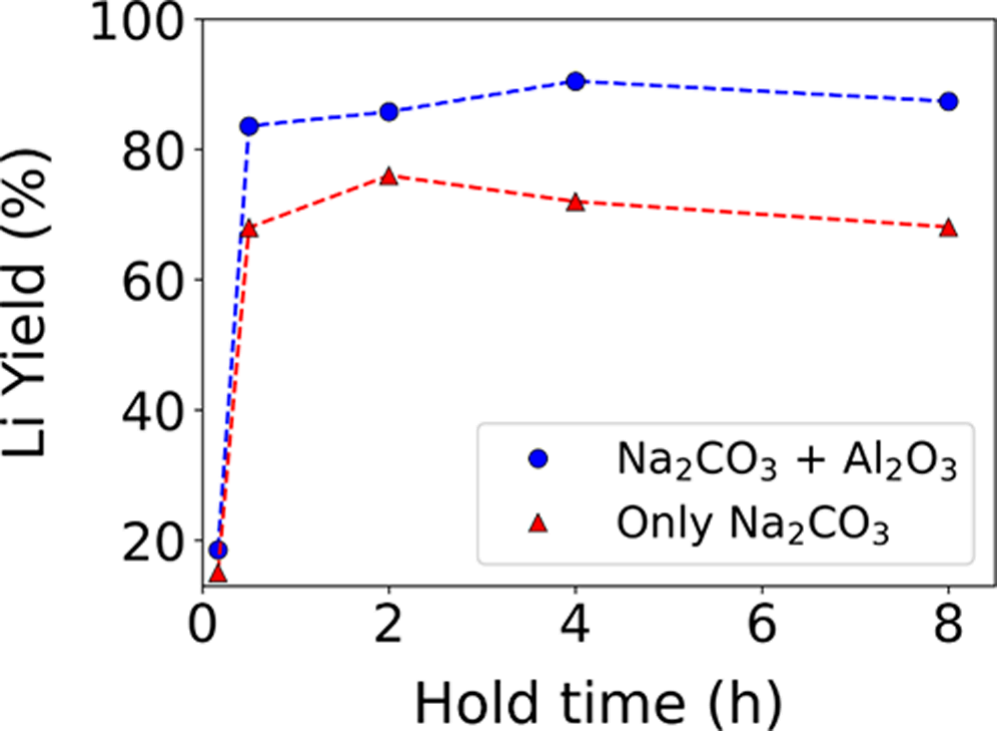
Percentage of Li extracted from spodumene in the form of Li_2_CO_3_ is plotted as a function of hold time at 750 °C for two different reactions (R1 and R2). These yields are determined from ICP-MS measurements performed on the leachate after washing. The blue circles and red triangles represent experimentally measured data whereas the dashed lines are linear interpolations.

To adjust the heating profile so that Li_2_CO_3_ is maximized, we tested various hold times at 750 °C for reactions with (R2) and without Al_2_O_3_ (R1). Five hold times were tested: 10 min, 30 min, 2, 4, and 8 h. After each hold, the sample was allowed to cool and Li_2_CO_3_ was separated from the reaction byproducts by washing the sample with water as described in previous sections. XRD patterns of the solid products were obtained for samples before and after washing. We also performed XRD on the leachate after evaporating water from it (Figures S7–S14). The yield of Li in the form of Li_2_CO_3_ was determined by ICP-MS (Table S1) as a percentage of the total Li available in spodumene. These values are plotted as a function of hold time for each set of reactants in Figure 5. Both reactions exhibit a similar trend in the Li yield, though adding Al_2_O_3_ (R2) consistently leads to higher yields (+15% on average). At a very short hold time of only 10 min, the Li yield ranges from 15% to 20% as the reaction with α-spodumene is likely incomplete. This reaction appears to progress quickly as a large increase in the Li yield is observed from 10 to 30 min, resulting in yields of 85% and 69% with and without Al_2_O_3_, respectively. At longer hold times, only small changes in the Li yield occur. Reactions involving Al_2_O_3_ reach a maximum yield of 90.4% at 4 h of hold time while those without Al_2_O_3_ reach a maximum yield of 76.4% at a shorter hold time of 2 h.

At hold times greater than 4 h, ICP-MS measurements indicate the Li yield tends to decrease for both reactions tested here. It is possible that this decrease can be attributed to the reaction that slowly occurs between Li_2_CO_3_ and SiO_2_; however, this seems unlikely as there is no clear change in the amount of Li_2_SiO_3_ formed at different hold times. Instead, we suspect that the observed decrease in Li yield is caused by the volatility of Li_2_CO_3_ at high temperature. Considering that Li_2_CO_3_ has a melting point of 732 °C,^25^ prolonged exposure at 750 °C is likely to result in some degree of volatility. This effect is well reported in previous studies, and as such, excess amounts of Li_2_CO_3_ are often used when synthesizing Li-ion cathodes at high temperature.^26−29^ For the extraction of Li from spodumene, we suggest that lower temperatures (≤750 °C) or relatively short hold times (≤4 h) should be used to avoid this volatility and therefore maximize the yield of Li_2_CO_3_.

### Process Overview

**Figure 6:**
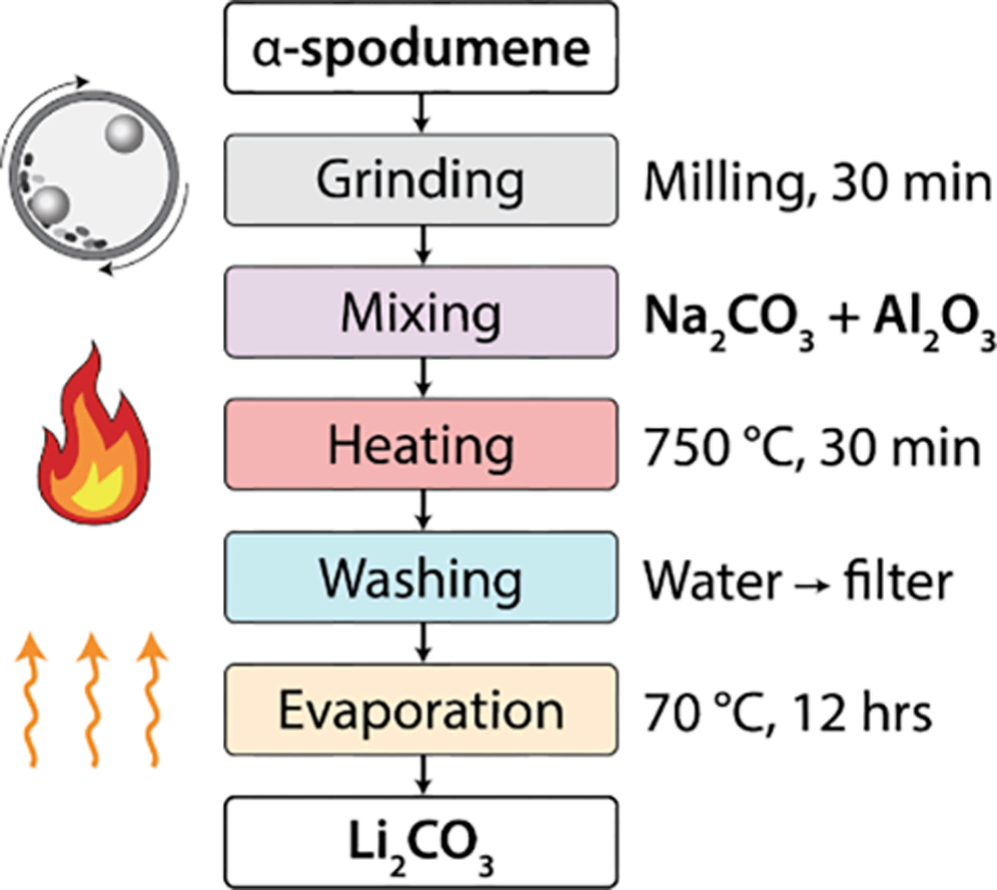
A workflow describing the optimized procedure for Li extraction from α-spodumene using solid-state reactions based on Na_2_CO_3_ and Al_2_O_3_.

Based on the findings reported in the previous few sections, we propose an optimized procedure (shown in Figure 6) for direct solid-state Li extraction from α-spodumene. The first step of this procedure involves grinding the spodumene concentrate to reduce particle size, thereby enhancing its reactivity to ensure that short annealing times can be used. The raw concentrate (without grinding) is less likely to react at an appreciable time scale given the presence of exceptionally large particles. After pulverization, the spodumene powder is mixed with Na_2_CO_3_ and Al_2_O_3_ in a molar ratio of 2:2:1 (LiAlSi_2_O_6_/Na_2_CO_3_/Al_2_O_3_), matching the reaction stoichiometry written in R2. The mixture is heated at 750 °C in air to form Li_2_CO_3_ and NaAlSiO_4_. The results shown in Figure 5 suggest that Li extraction is almost completed after 30 min of holding at 750 °C, resulting in a high Li_2_CO_3_ yield of 85%. Even higher yield (up to 90.4%) can be achieved when using a longer hold time of 4 h. The optimal hold time can be determined by the desired trade-off between energy consumption and Li yield. After completing the solid-state reaction, Li_2_CO_3_ can be separated from the byproducts (mostly NaAlSiO_4_ and Li_2_SiO_3_) by washing the sample with water and filtering it through an inorganic membrane. Evaporation of water from the leachate at 70 °C leads to the precipitation of Li_2_CO_3_. In principle, the Li yield could be further increased by removing any Li in the silicate byproducts through a secondary acid leaching procedure.^13^

### Discussion

Spodumene is the resource that currently accounts for about 50% of all Li used in battery cathode materials production,^30^ and its market share is growing. Sourcing Li from spodumene benefits from the fact that it is often refined at a much faster rate than from brines, which can take months or even years to evaporate.^4^ However, extracting Li from spodumene tends to be more costly and energy-intensive owing to the high temperature (>1000 °C) that is typically considered as a necessary step to transform α-spodumene to its lower-density β polymorph for enhanced acid leaching efficiency. In this work, we proposed an alternative approach whereby α-spodumene is reacted directly with Na_2_CO_3_ and Al_2_O_3_ at 750 °C for short duration, resulting in the successful extraction and recovery of Li in the form of Li_2_CO_3_. We have demonstrated that Li_2_CO_3_ can be separated from other reaction byproducts (e.g., NaAlSiO_4_ and Li_2_SiO_3_) by washing the mixture with deionized water, thus leveraging the differences in their solubilities. This process leads to a high 90.4% yield of Li after only 4 h of heating time, as evidenced by ICP-MS performed on the leachate.

The findings presented here build upon important contributions from recent efforts, which proposed Na_2_CO_3_ as an effective reactant for the extraction of Li from spodumene in the solid state.^12,14^ Our work further reveals that introducing Al_2_O_3_ as a second reactant can boost the Li yield by as much as 14%. The addition of Al_2_O_3_ not only leads to a near 4-fold increase in the reaction’s driving force, but also reduces the formation of Li_2_SiO_3_. While the solid reactants used here generally cost more than acids that would otherwise be used for Li refinement, there is opportunity to further reduce costs by using other forms of Al (besides Al_2_O_3_) that are more common mineralogically. For example, AlOOH (boehmite), Al(OH)_3_ (gibbsite) and their mixtures in bauxite could be used as they will likely decompose to Al_2_O_3_ before reacting with spodumene. Furthermore, solid reactants are easier to handle and store as opposed to acids, while alleviating the risk of instrument corrosion.

Our process is beneficial as it does not require a high temperature heating step to transform spodumene from its α to β polymorph, thereby reducing the total energy input. All operations involved in the proposed method are readily scalable and could lead to substantially reduced costs at the industrial scale. Furthermore, we completely avoid the need for acid wash, instead relying only on water to separate Li_2_CO_3_ from the byproducts. By eliminating the use of acid, the risk of equipment corrosion is alleviated, which can assist in reducing the required facility maintenance costs. Following the washing procedure used to isolate Li_2_CO_3_, we are left with NaAlSiO_4_ as a primary byproduct. This material may be used for glass manufacturing (in the place of feldspar) or as a filler in paints, plastics, foam rubbers, and sorbents.^31^ The findings presented in this work demonstrate the feasibility of spodumene as a low-cost Li source, which can increase the adoption of domestically sourced ores for cathode production. Our work also shows that α-spodumene itself can be used as an effective precursor, which opens new opportunities for the direct synthesis of battery materials from raw ores.

## Conclusions

In summary, we have demonstrated that Li can be extracted directly from the low-temperature (α) polymorph of spodumene by reacting it with Na_2_CO_3_ and Al_2_O_3_. The use of Na_2_CO_3_ provides a large thermodynamic driving force to extract Li in the form of Li_2_CO_3_ while simultaneously producing NaAlSiO_4_ and SiO_2_. Because the presence of SiO_2_ in the reaction mixture can lead to unwanted Li_2_SiO_3_ formation, Al_2_O_3_ is added with excess Na_2_CO_3_ to completely trap the silicate anion in NaAlSiO_4_, thereby reducing SiO_2_ (and subsequent Li_2_SiO_3_) formation. Through this process, as much as 90.4% of Li can be extracted from spodumene within only 4 h of heating at 750 °C. Isolation of Li_2_CO_3_ can be achieved through a washing procedure that only uses water, circumventing the need for acids. Our work shows that Li extraction from spodumene is possible without requiring a high-temperature (>1,000 °C) annealing step to transform the material into its more reactive β polymorph. It may therefore be possible to modify the current approaches to Li refinement and satisfy the growing demand for Li_2_CO_3_ production at lower cost.

## Methods

### Material Preparation and Synthesis

The lithium-based ore we used was α-spodumene (LiAlSi_2_O_6_), obtained from deposits located in North Carolina, United States. The spodumene concentrate was ground and sieved to a particle size less than 75 μm. The processed spodumene samples were combined with Na_2_CO_3_ (Sigma-Aldrich, 99.5%) and Al_2_O_3_ (Sigma-Aldrich, nanopowder) as precursors for Li extraction. These precursors were stoichiometrically mixed, with 33% excess spodumene added to compensate for the impurities that accompany it, using a Retsch PM 200 planetary ball mill at 250 rpm for 12 h. The precursors were then dried in a 70 °C oven overnight and pelletized. The precursors were sintered at 750 °C in air for different times (10 min, 30 min, 2, 4, and 8 h) before letting them cool naturally to room temperature.

### Separation

The as-synthesized samples were manually ground with mortar and pestle into fine powders and then added into deionized water, followed by sonication for 1 h. The solutions were stirred for 1.5 h to fully dissolve Li_2_CO_3_. Suction filtration was performed to separate the liquid from the solid. The resulting solid was dried in a 70 °C vacuum oven, while the liquid was dried in a 70 °C convection oven. Both samples were held overnight to ensure evaporation of the water. XRD measurements were performed on both the solid residue (not dissolved in water) and the solid dried from the leachate. ICP-MS measurements were conducted only on the leachate.

### X-ray Diffraction

In-house XRD patterns were obtained using a Rigaku MiniFlex and Aeris benchtop diffractometer (each with a Cu Kα source). *In situ* XRD was performed using beamline 12.2.2 at the Advanced Light Source (ALS) of Lawrence Berkeley National Laboratory (LBNL), using radiation with a constant energy of 25 keV. All *in situ* samples were packed into sapphire capillaries before exposing them to the X-rays and heated to 800 °C at a ramp rate of 10 °C/min under air, without any gas flow. A diffraction pattern was acquired once every 15 s during heating and one every 30 s during the hold. Calibration was performed using a lanthanum hexaboride (LaB_6_) standard. Rietveld refinement was used to estimate the weight fraction of any crystalline phases detected in XRD; however, this method was not used for quantitative analysis of the Li yield. ICP-MS was instead used for that purpose, as described in the next section.

### Elemental Analysis

The spodumene concentrate provided by Piedmont Lithium Inc. was first ground into powder. Acids of HNO_3_, HCl and HF were successively used to digest the mineral. HF breaks down the silicates and produces SiF_4_, which becomes volatile and is lost when the samples is dried. During the dry down stage, perchloride acid was added to break down any other fluorides that might have formed. The concentrations of different elements were determined by Agilent HPLC-ICP-MS measurements at the geochemical and analytical facility of Earth and Environmental Sciences Area (EESA) of LBNL. Two measurements were conducted and the results were consistent with one another. The average concentrations of these measurements are reported main text (Table 2). Since SiF_4_ is volatile, the SiO_2_ concentration was calculated based on mass balance. Elemental analysis of the leachate after filtration was performed at LBNL using an Agilent 7900 ICP-MS. The calibration curve was generated using standard solutions with 5 different concentrations ranging from 1 to 1000 ppb, and linear fitting was applied. All solutions were diluted to concentrations between 1 to 1000 ppb using a 5% nitric acid solution.

### Calculation of Reaction Energies

To compute the energy change associated with each reaction, we followed a similar procedure to that outlined in previous work.^32^ The formation energy of each solid reactant and product was extracted from the Materials Project,^33^ which computed these energies using density functional theory (DFT) calculations based on the r^2^SCAN functional.^34^ Finite temperature effects were accounted for by using a machine-learned descriptor of the vibrational entropy developed by Bartel et al.^35^ All phases were assumed to be ordered, and as such, no configurational entropy was accounted for. The zero-temperature (0 K) formation energies computed by DFT were summed with the finite temperature corrections to obtain the Gibbs free energy of each phase, neglecting any effects of pressure which are small for most solids. The energy change associated with each reaction was then calculated by taking the difference between the Gibbs free energy of the products and reactants at the temperature where that reaction was observed. The resulting energies are normalized by the total number of atoms in the products formed.

## Data Availability

All the data presented in this work can be found in the published article and its Supporting Information.

## Supporting Information Available

Additional characterization results including SEM images of spodumene, Rietveld refinements, XRD measurements performed at varied reaction conditions, and ICP-MS results (PDF)
