# Supplementary material for: Direct Lithium Extraction from α-Spodumene through Solid-State Reactions for Sustainable Li2CO3 Production
Source: Inorg Chem. 2024 Jul 9;63(29):13576–84. doi: 10.1021/acs.inorgchem.4c01722 (PMC11270991; doi:10.1021/acs.inorgchem.4c01722)
Supplement: Supplementary file 1 — ic4c01722_si_001.pdf [file ic4c01722_si_001.pdf]

Supplementary information

Direct lithium extraction from α-spodumene through solid-state reactions for sustainable Li_2CO_3 production

Shilong Wang[1,2], Nathan J. Szymanski[1,2], Yuxing Fei[1,2], Wenming Dong[3], John N. Christensen[3], Yan Zeng[2,4,*], Michael Whittaker[3,5], and Gerbrand Ceder[1,2,*]

[1] Department of Mat. Sci. & Engineering, UC Berkeley, Berkeley, CA 94720, USA
[2] Materials Sciences Division, Lawrence Berkeley National Laboratory, Berkeley, CA 94720, USA
[3] Energy Geosciences Division, Lawrence Berkeley National Laboratory, Berkeley, CA 94720, USA
[4] Department of Chemistry & Biochemistry, Florida State University, Tallahassee, FL 32306, USA
[5] Department of Earth & Planetary Sci., UC Berkeley, Berkeley, CA 94720, USA
[*] Corresponding authors, zeng@chem.fsu.edu (Y. Z.) and gceder@berkeley.edu (G. C.)

Information included:

Figures S1-S14

Table S1

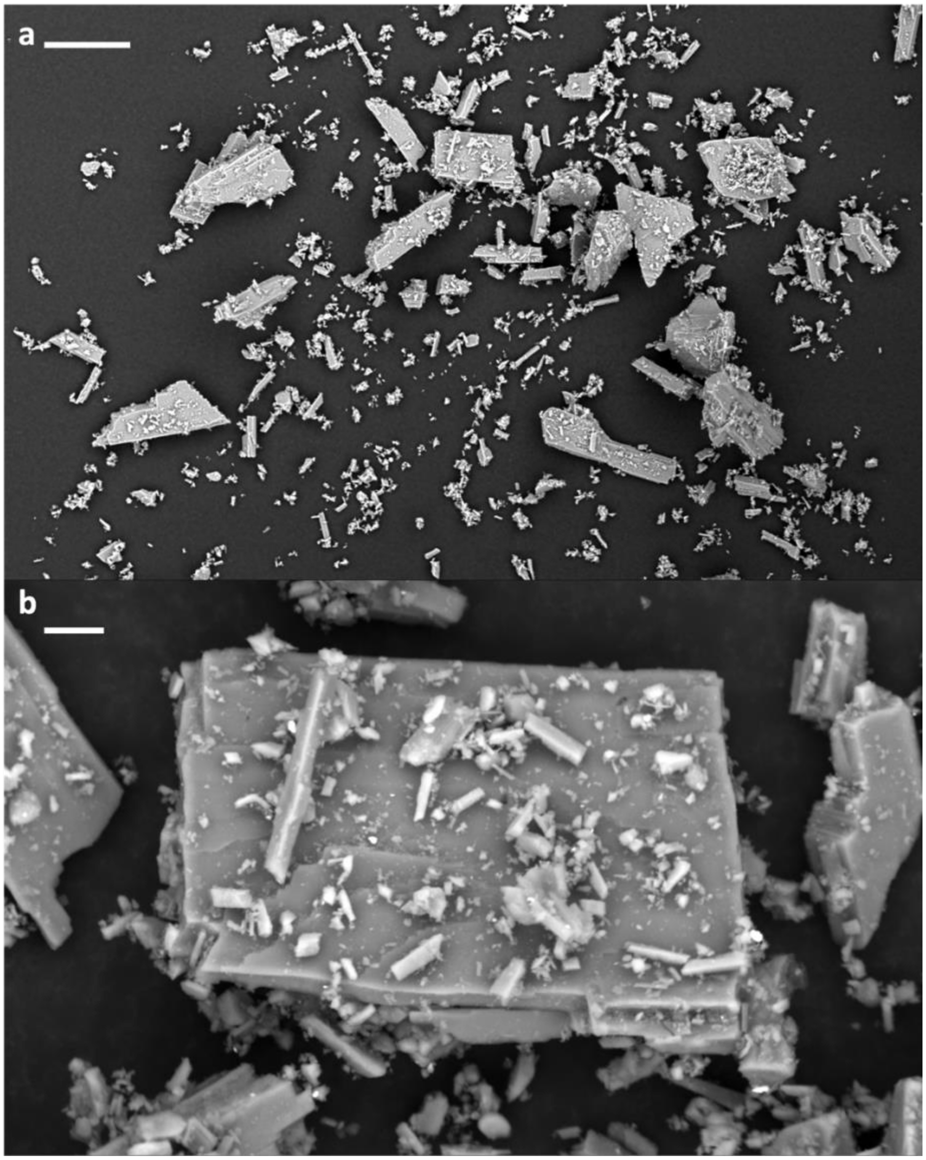

Figure S1. a) SEM image collected from the spodumene concentrate sample after grinding and filtration. b) Enlarged view of a particular region from a). The scale bar in the top panel represents 50 µm, while the scale bar in the bottom panel represents 5 µm. Both images were obtained from a Phenom XL Desktop SEM from Thermo Fisher Scientific, operated at a voltage of 15 kV with a pressure of 0.1 Pa.

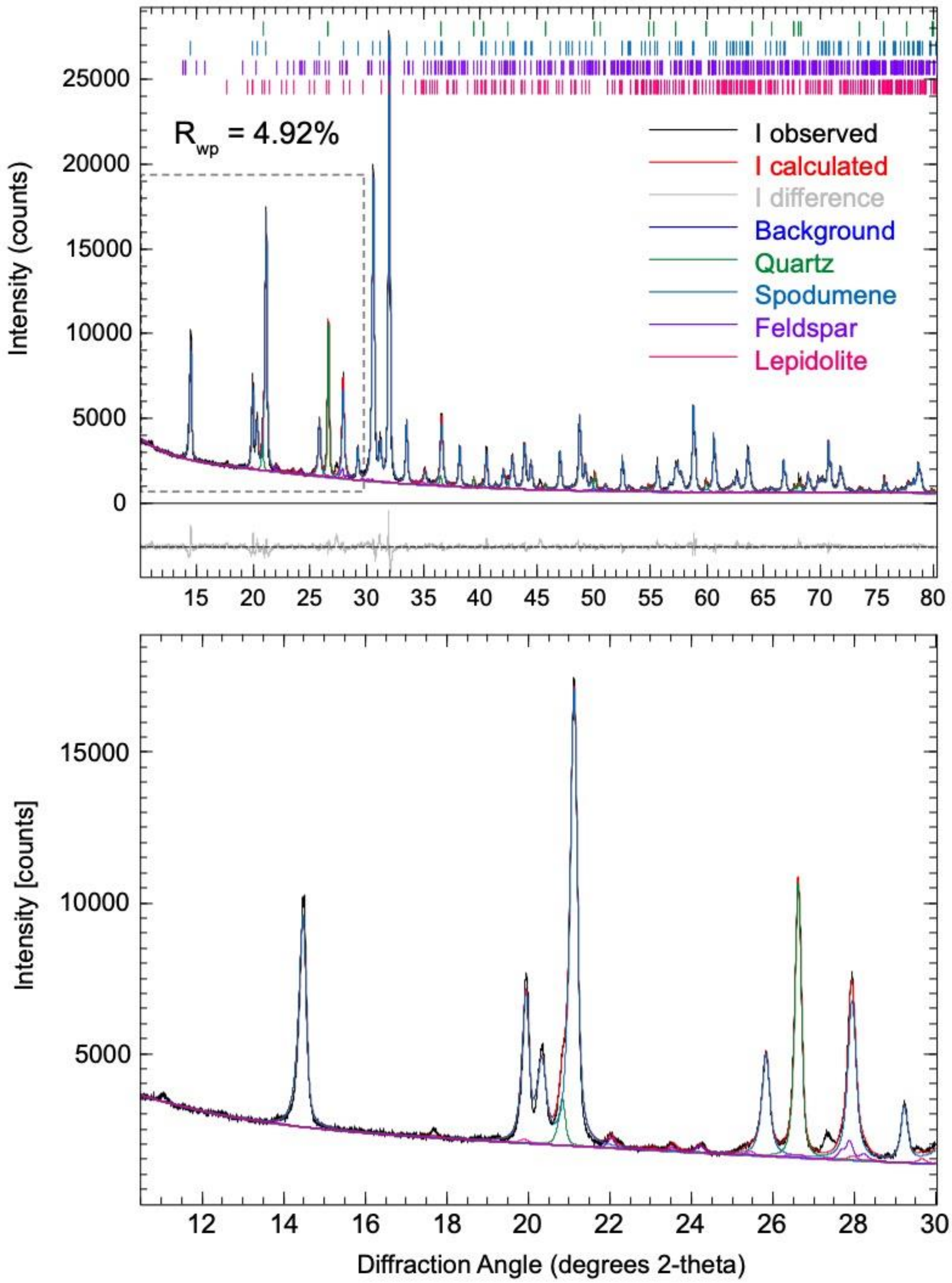

Figure S2. Rietveld refinement performed using an XRD pattern collected from spodumene concentrate after grinding. The bottom panel shows a magnified version of the pattern in the top panel, focusing on the low-angle regime.

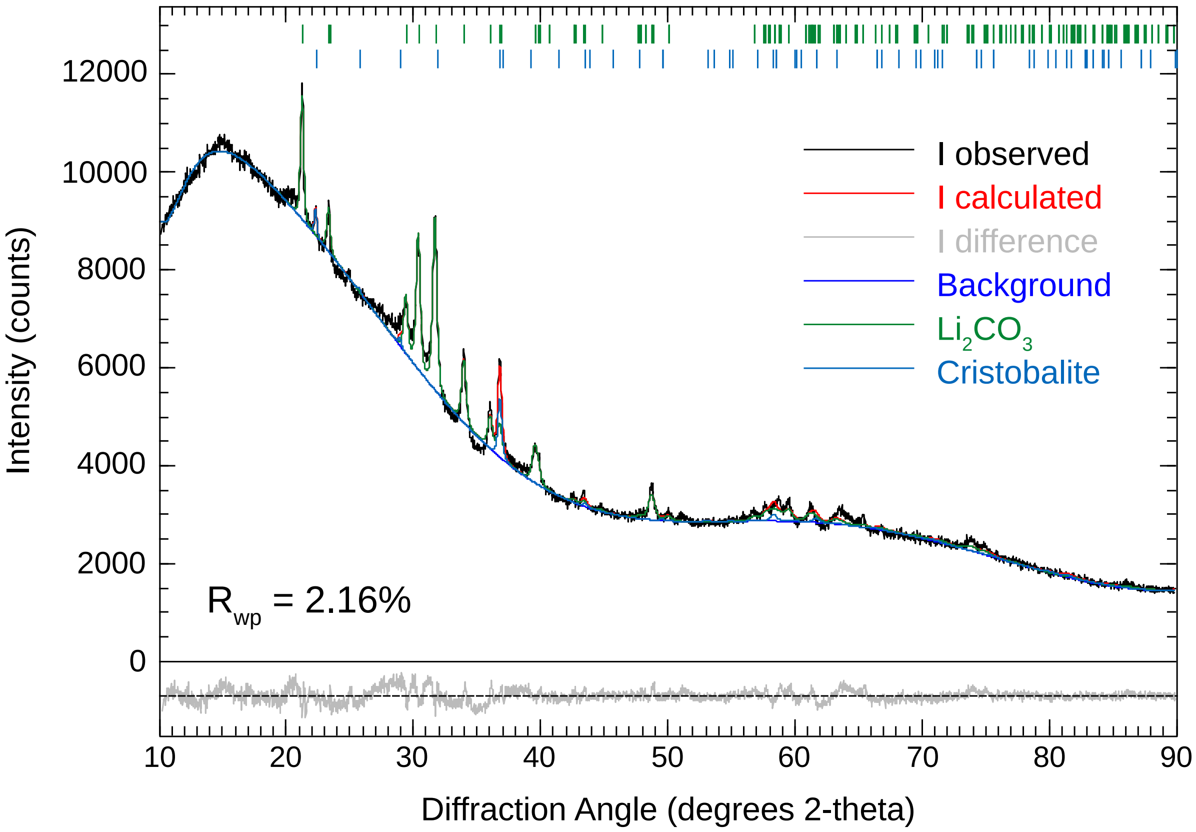

Figure S3. Rietveld refinement performed using an XRD pattern collected from the solid obtained after drying the leachate that results from the synthesis products of Na_2CO_3 and $NaAlSiO_4$ (750 °C, 30 min).

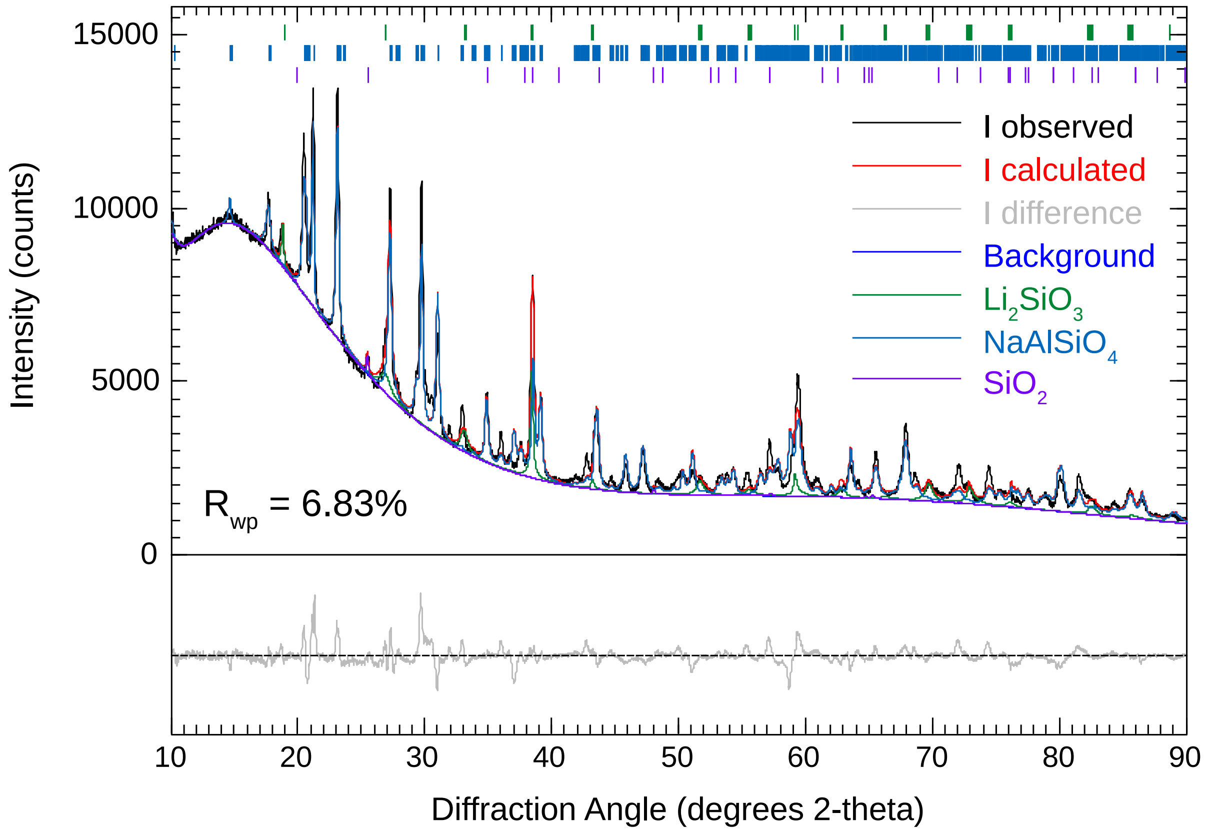

Figure S4. Rietveld refinement performed using an XRD pattern collected from the solid residue that remains after washing the synthesis products of Na_2CO_3 and spodumene (750 °C, 30 min). The SiO_2 peaks correspond to its high-temperature (β) polymorph.

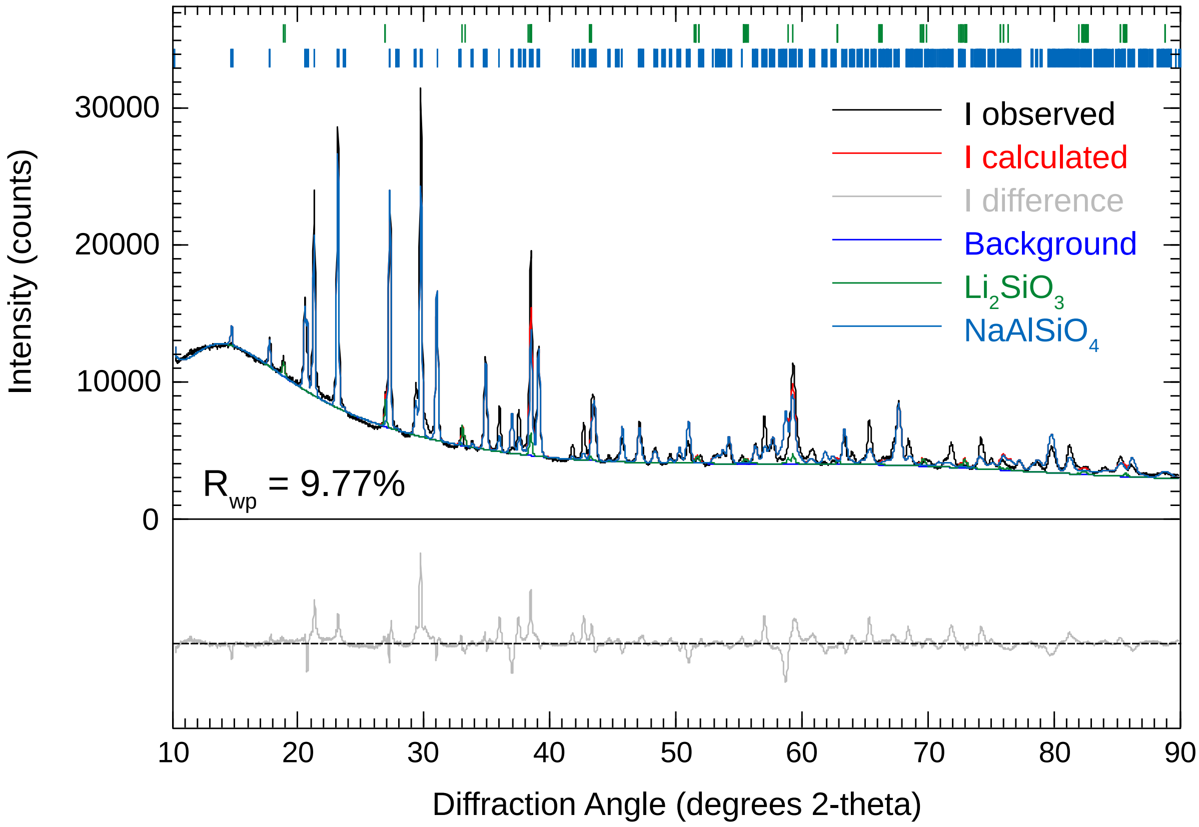

Figure S5. Rietveld refinement performed using an XRD pattern collected from the solid residue that remains after washing the synthesis products that result from reacting spodumene with Na_2CO_3 and Al_2O_3 (750 °C, 30 min). We do not detect any SiO_2 in this pattern, possibly due to a reaction with the Al_2O_3 precursor to form an aluminum silicate.

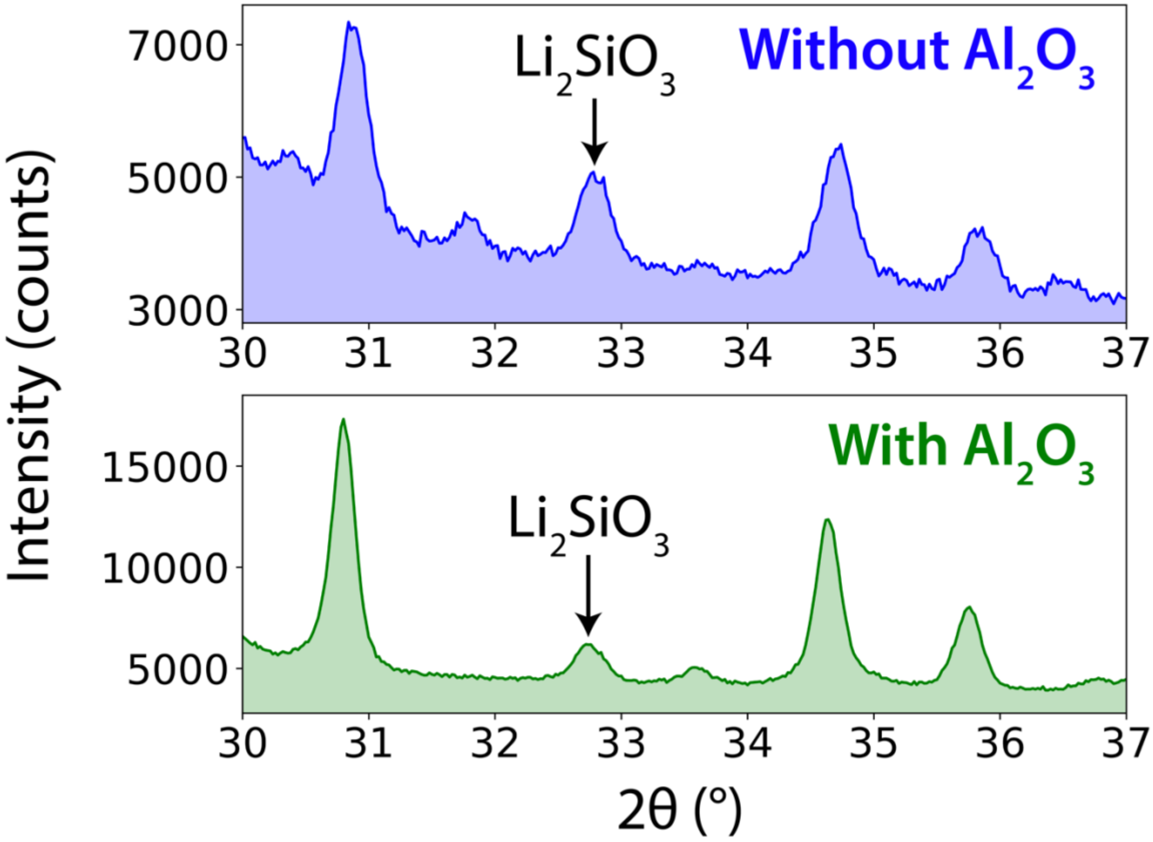

Figure S6. Portions of the XRD patterns collected from the solid residue that remains after washing the synthesis products that result from reacting spodumene with Na_2CO_3, either with Al_2O_3 (bottom panel) or without Al_2O_3 (top panel). Both samples were held at 750 °C for 30 min before letting them cool. This portion of the XRD pattern highlights the decrease in Li_2SiO_3 that occurs when Al_2O_3 is used as an additive.

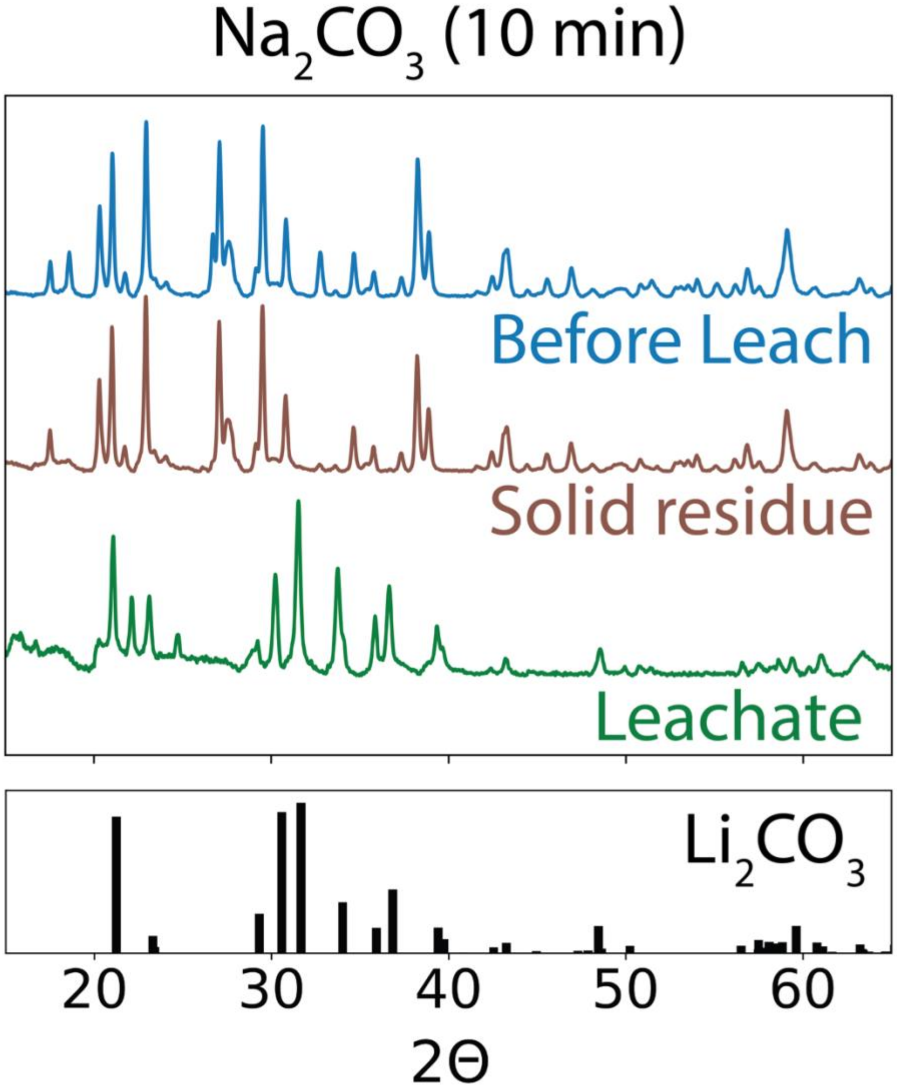

Figure S7. XRD patterns of the products obtained by reacting α-spodumene with Na_2CO_3 for 10 minutes. The data is shown for the sample before and after leaching with water. The washing procedure results in a solid residue (not dissolved) and a leachate which is dried and characterized. The XRD pattern of this leachate confirms the presence of Li_2CO_3 (ICSD #66941), whose reference pattern is shown in the lower panel.

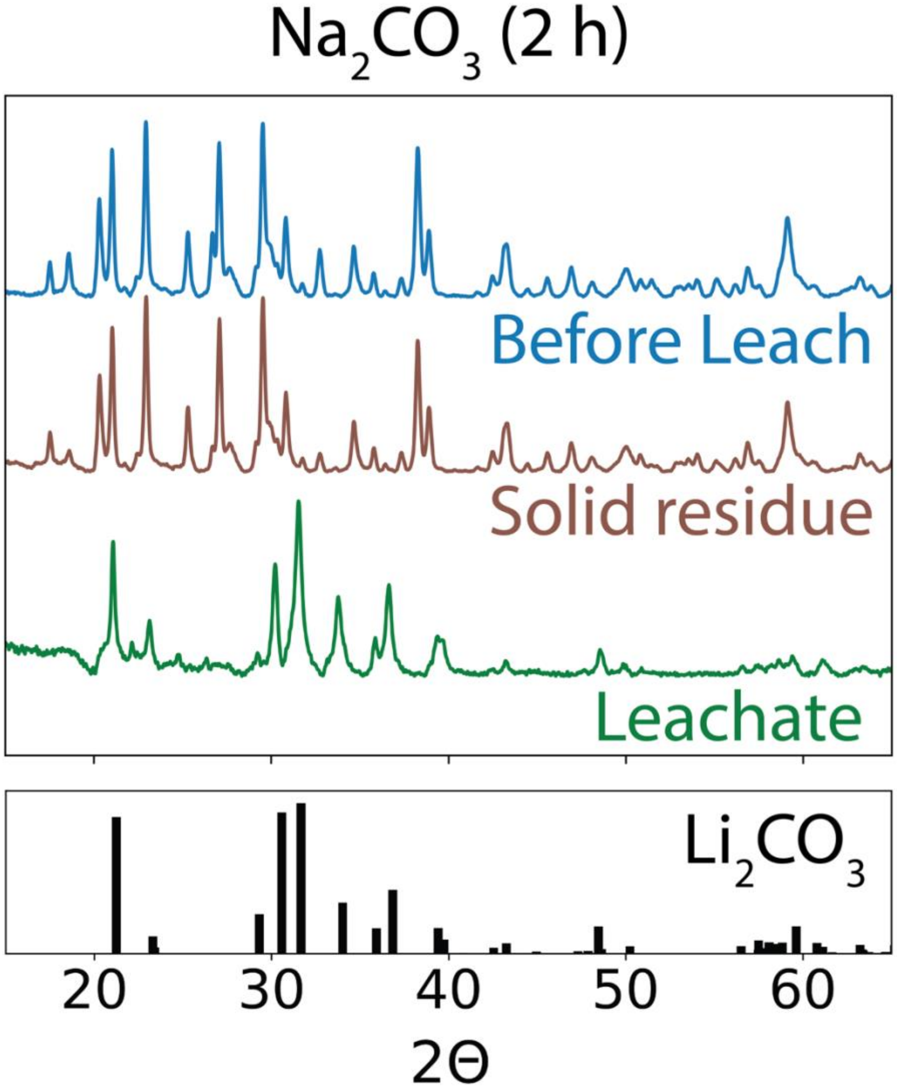

Figure S8. XRD patterns of the products obtained by reacting α-spodumene with Na_2CO_3 for 2 hours. The data is shown for the sample before and after leaching with water. The washing procedure results in a solid residue (not dissolved) and a leachate which is dried and characterized. The XRD pattern of this leachate confirms the presence of Li_2CO_3 (ICSD #66941), whose reference pattern is shown in the lower panel.

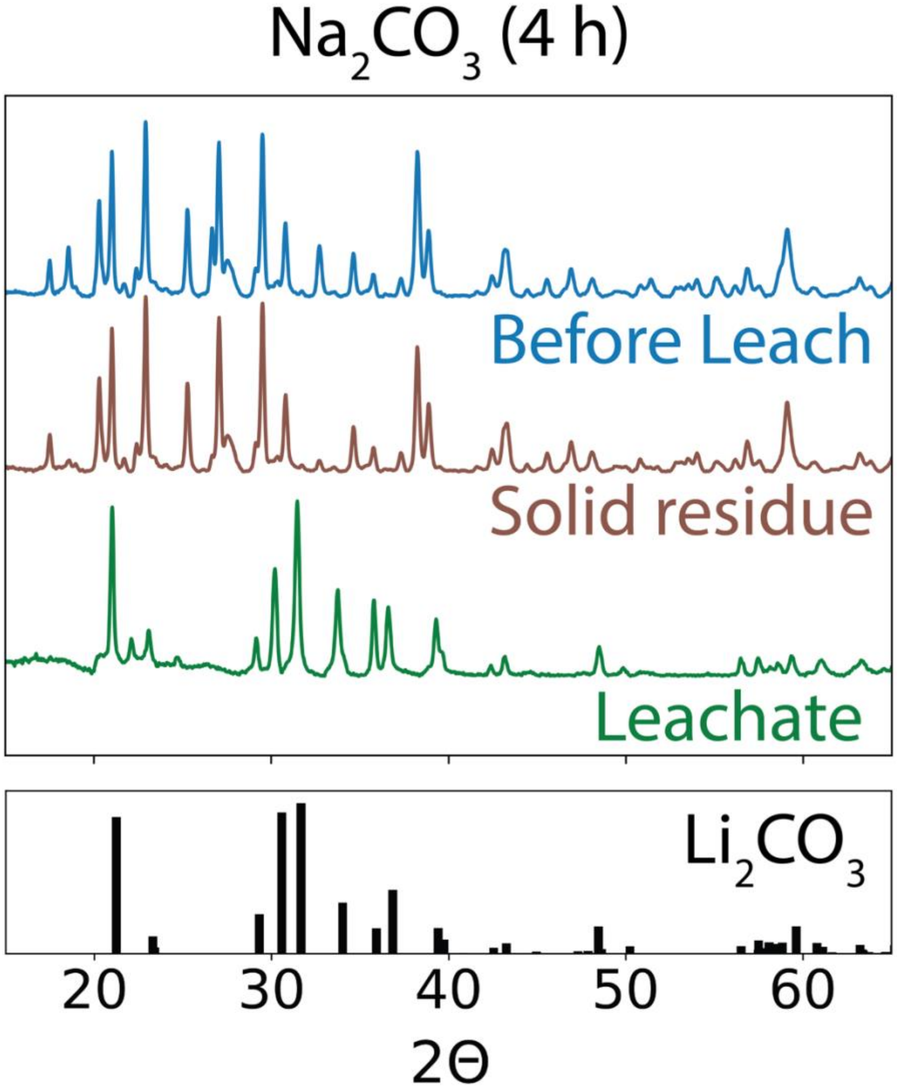

Figure S9. XRD patterns of the products obtained by reacting α-spodumene with Na_2CO_3 for 4 hours. The data is shown for the sample before and after leaching with water. The washing procedure results in a solid residue (not dissolved) and a leachate which is dried and characterized. The XRD pattern of this leachate confirms the presence of Li_2CO_3 (ICSD #66941), whose reference pattern is shown in the lower panel.

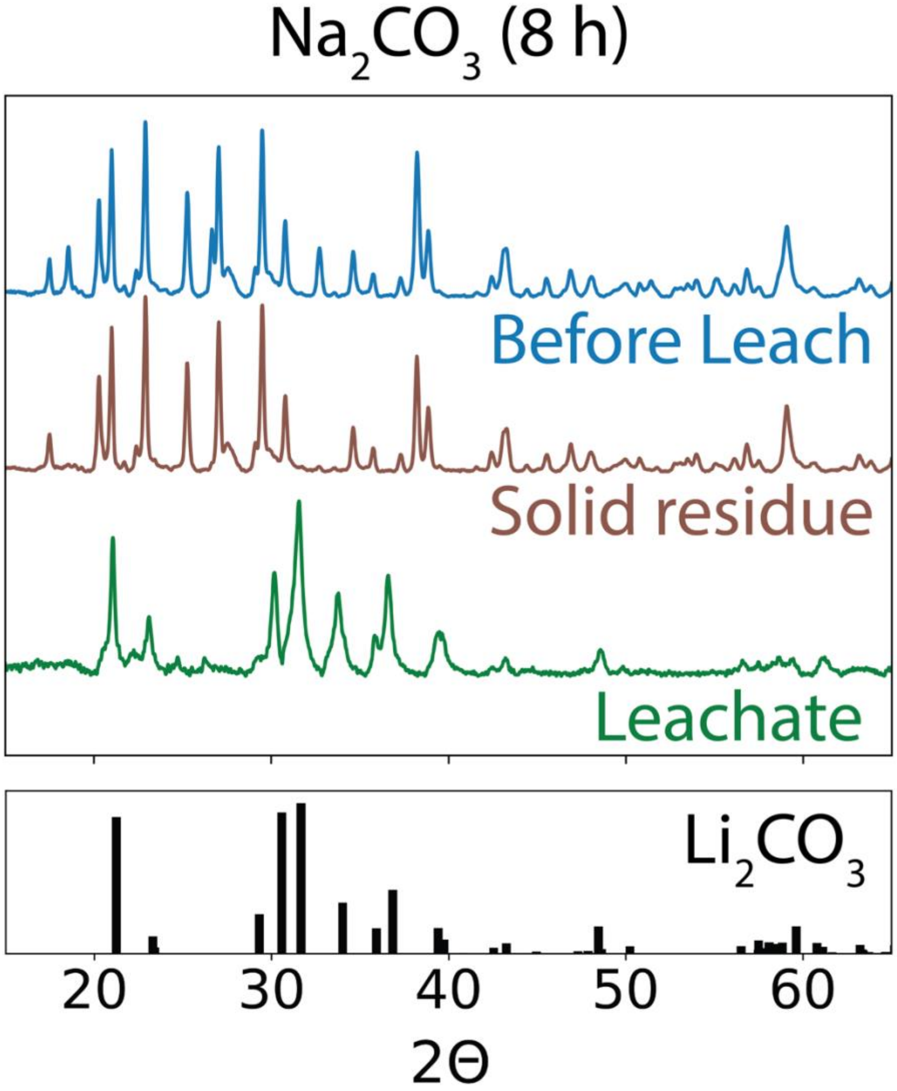

Figure S10. XRD patterns of the products obtained by reacting α-spodumene with Na_2CO_3 for 8 hours. The data is shown for the sample before and after leaching with water. The washing procedure results in a solid residue (not dissolved) and a leachate which is dried and characterized. The XRD pattern of this leachate confirms the presence of Li_2CO_3 (ICSD #66941), whose reference pattern is shown in the lower panel.

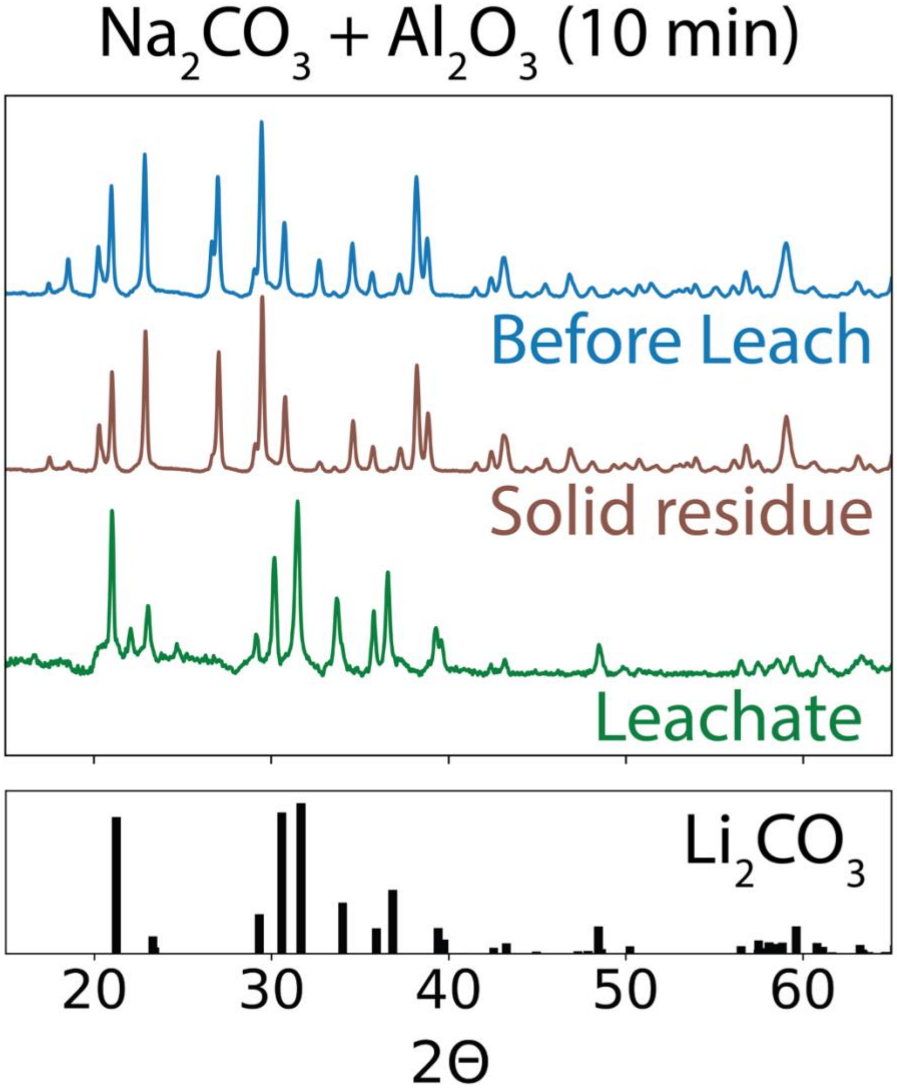

Figure S11. XRD patterns of the products obtained by reacting α-spodumene with Na_2CO_3 and Al_2O_3 for 10 minutes. The data is shown for the sample before and after leaching with water. The washing procedure results in a solid residue (not dissolved) and a leachate which is dried and characterized. The XRD pattern of this leachate confirms the presence of Li_2CO_3 (ICSD #66941), whose reference pattern is shown in the lower panel.

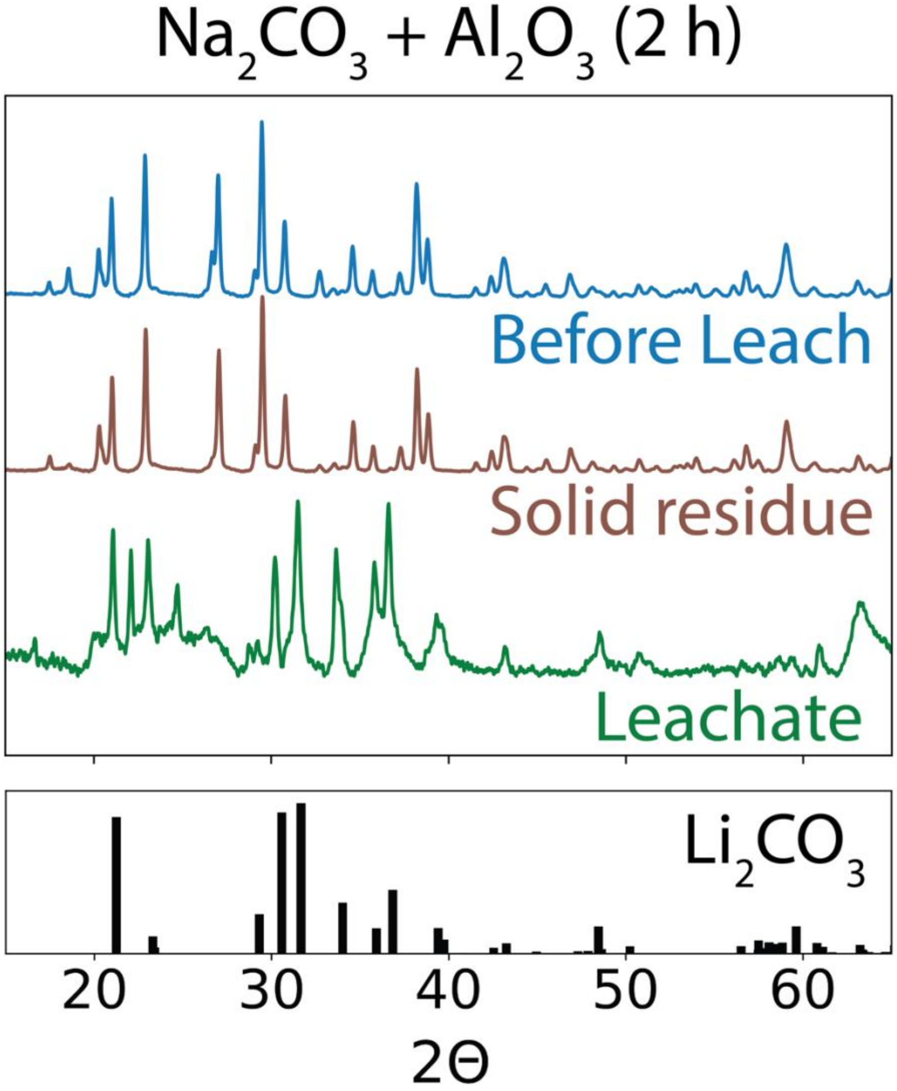

Figure S12. XRD patterns of the products obtained by reacting α-spodumene with Na_2CO_3 and Al_2O_3 for 2 hours. The data is shown for the sample before and after leaching with water. The washing procedure results in a solid residue (not dissolved) and a leachate which is dried and characterized. The XRD pattern of this leachate confirms the presence of Li_2CO_3 (ICSD #66941), whose reference pattern is shown in the lower panel.

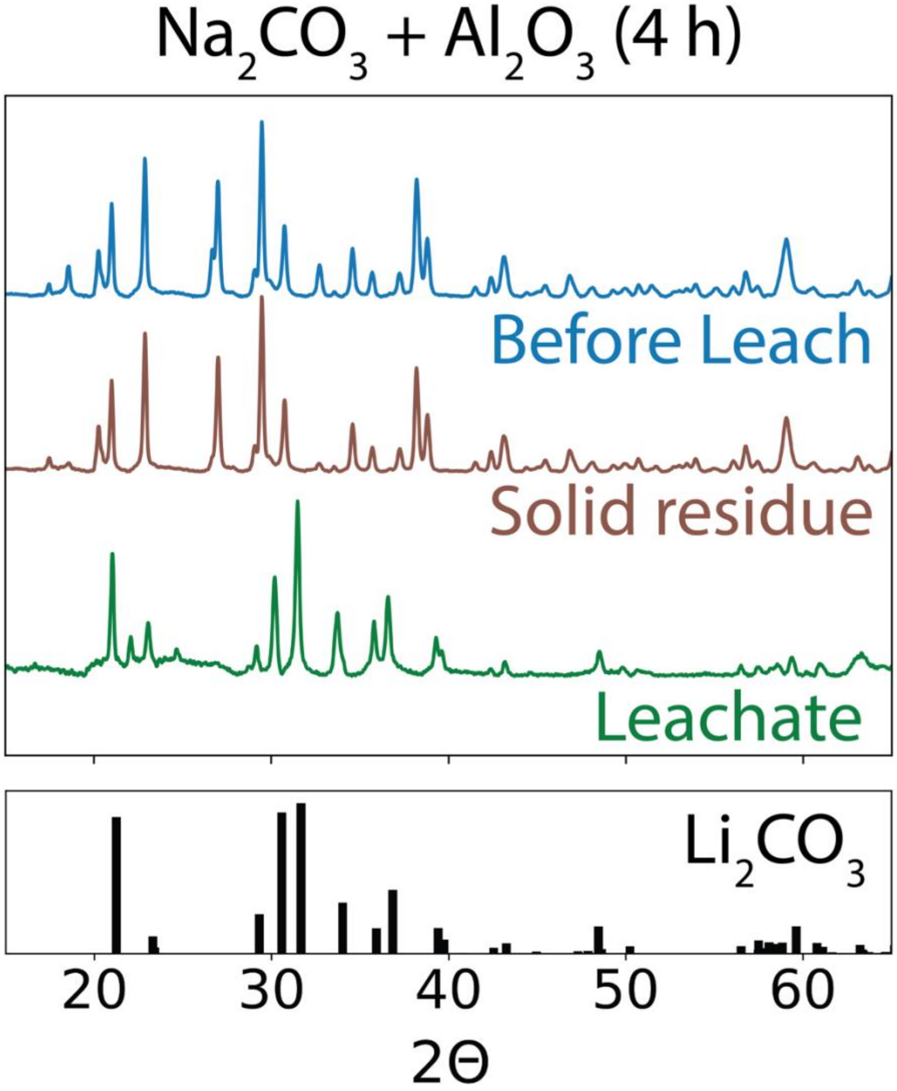

Figure S13. XRD patterns of the products obtained by reacting α-spodumene with Na_2CO_3 and Al_2O_3 for 4 hours. The data is shown for the sample before and after leaching with water. The washing procedure results in a solid residue (not dissolved) and a leachate which is dried and characterized. The XRD pattern of this leachate confirms the presence of Li_2CO_3 (ICSD #66941), whose reference pattern is shown in the lower panel.

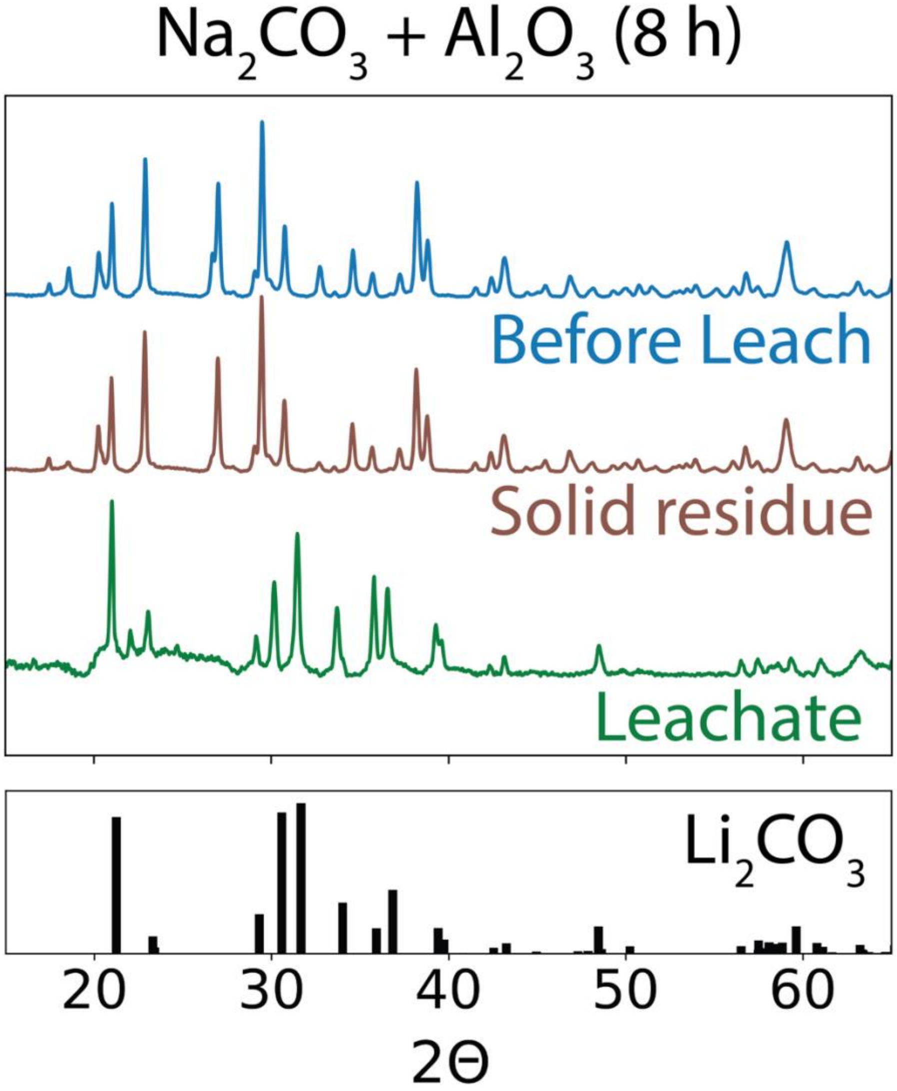

Figure S14. XRD patterns of the products obtained by reacting α-spodumene with Na_2CO_3 and Al_2O_3 for 8 hours. The data is shown for the sample before and after leaching with water. The washing procedure results in a solid residue (not dissolved) and a leachate which is dried and characterized. The XRD pattern of this leachate confirms the presence of Li_2CO_3 (ICSD #66941), whose reference pattern is shown in the lower panel.

Table S1. Elemental analysis of the leachate after using different combinations of precursors for Li extraction from spodumene. These were determined using the ICP-MS measurements described in the Methods of the main text.

Precursors	Hold time	Product weight (mg)	Lithium concentration after dilution (ppb)	Extraction efficiency (%)
2 Spodumene + Na_2CO_3	10 minutes	702.1	12.7265	15.32
	30 minutes	300.35	27.9347	68.69
	2 hours	300.07	31.0028	76.41
	4 hours	300.80	29.4670	72.45
	8 hours	299.70	27.6276	68.18
2 Spodumene + 2 Na_2CO_3 + Al_2O_3	10 minutes	727.7	12.5491	18.54
	30 minutes	300.79	27.0279	84.55
	2 hours	300.22	27.0925	84.91
	4 hours	300.95	28.9288	90.45
	8 hours	299.64	27.8233	87.37
